# Serum ferritin levels in a sample of older participants in the Greek HYDRIA survey: associations with sociodemographic and lifestyle parameters including dietary iron intake and Mediterranean diet score

**DOI:** 10.1017/S0007114526106540

**Published:** 2026-06-14

**Authors:** Eleni Maria Papatesta, Georgia Vourli, Eleni Peppa, Ioannis Patsis, Ariadne Malamitsi-Puchner, Antonia Trichopoulou

**Affiliations:** 1 Center for Public Health Research and Education, Academy of Athens, Alexandroupoleos 23, Athens 11528, Greece; 2 School of Medicine, National and Kapodistrian University of Athens, 75 Mikras Asias Str., Athens 11527, Greece

**Keywords:** Iron deficiency, Ferritin, Mediterranean diet score, HYDRIA, Dietary iron intake

## Abstract

Iron deficiency (ID) represents the most prevalent nutritional disorder and a major public health concern. As part of the HYDRIA 2013–2014 National Health and Nutrition study, a cross-sectional survey of a nationally representative sample of adults in Greece, serum ferritin levels (SFL) were measured in participants aged ≥ 55 years, and dietary intake was assessed using two non-consecutive 24-h dietary recalls per participant. The study aimed to evaluate SFL, determine the prevalence of ID among older adults and explore associations with dietary iron intake and adherence to the Mediterranean diet (MD). Among individuals over 55 years, ID prevalence was 6·5 % for SFL < 15 ng/ml and 12·7 % for SFL < 30 ng/ml. SFL were significantly associated with sex and age (*P* < 0·001), with men showing higher SFL than women. Advancing age, particularly among individuals over 74 years, increased the risk of ID. Dietary iron intake declined with age, and women had significantly lower intake than men (*P* < 0·001). Overall, 57·2 % of participants had inadequate daily iron intake. SFL were not associated with total or haem iron intake. High adherence to MD was associated with higher energy-adjusted total iron intake but lower haem iron intake. Weighted logistic regression for SFL < 15 ng/ml showed that MD adherence was significantly associated with reduced odds of ID (*P* = 0·007), likely due to the protective effect of intermediate adherence (OR = 0·10, *P* = 0·002). Conclusively, while ID prevalence among older adults in Greece is low, over half of the population presents inadequate daily iron intake, especially women. High adherence to MD may have a protective effect against ID.

Among micronutrients, iron plays a pivotal role in many pathophysiologic and cell processes such as the transportation of oxygen, Hb formation, DNA synthesis and energy generation^(1)^. Iron deficiency (ID) is the most common nutritional deficiency and a public health matter of great importance. It is related to several causes, including insufficient intake and poor absorption, and is associated with physical, cognitive and immune impairment^(1,2)^. The number of ID cases worldwide increased from 984·61 million in 1990 to 1270·64 million in 2021, and by 2050, it is expected to reach 1439·99 million. Disability-adjusted life years from ID were 32·32 million in 2021 and are expected to reach 36·13 million by 2050^(3)^. ID is one of the main causes of anaemia (ID anaemia)^(4)^, especially for high-risk populations such as the older population^(5)^. In older individuals, malnutrition, impaired absorption, coexisting chronic diseases and medication may lead to ID, which in turn has been linked to all-cause mortality and morbidity^(5,6)^. Thus, identifying older adults as a high-risk group for ID, as well as dietary and lifestyle factors that contribute to this risk, is crucial. Serum ferritin levels (SFL) are the most specific indicator for the diagnosis of ID, as proposed by the majority of guidelines, albeit suggested cutoffs differ^(7)^. According to the WHO, a cutoff of SFL < 15 ng/ml is recommended for the diagnosis of ID, yet additional cutoffs are used in everyday practice such as SFL < 30 ng/ml^(2,7)^. SFL, as an acute-phase protein, rises during inflammation, and age-related chronic low-grade inflammation (‘inflammaging’) can obscure its portrayal of iron status^(8)^. Therefore, the WHO recommends measuring an additional acute-phase marker, such as C-reactive protein (CRP), alongside SFL, with various adjustment methods proposed^(2)^.

Traditionally, the Greek population adhered to the Mediterranean diet (MD), exerting a beneficial effect on health^(9)^. MD contains several iron chelators, which bind to intracellular labile iron, thus protecting the cells from oxidative stress^(10)^. However, concerns have been expressed that despite higher iron intakes from an MD^(11)^, these may not necessarily result in higher iron status, due to restricted consumption of red meat, important for bioavailable iron. Additionally, phytate – which is found in whole-grain cereals, an important constituent of MD – inhibits iron absorption, possibly resulting in lower iron status^(12)^.

The purpose of this secondary analysis of the HYDRIA study is to assess SFL, recognised as the most commonly used marker of iron stores^(7,13)^, and to determine the prevalence of ID in the older adult population in Greece and its association with several sociodemographic and lifestyle factors, including dietary iron intake and adherence to MD.

## Material and methods

### Study design and population

HYDRIA is a large nationally representative Greek survey that investigated food, macronutrient and micronutrient intakes in a sample of 4011 adults over 18 years of age, providing observations and conclusions on population health in relation to the nutritional status of the population. Field data were collected between June 2013 and December 2014. The survey was conducted in accordance with the guidelines set by the Helsinki Declaration and national legislation regarding data protection. The methodology of HYDRIA was based on the recommendations of the European Food Safety Authority (EFSA)^(14)^ and the European Health Examination Survey^(15)^. The HYDRIA survey characteristics, sampling methods, inclusion criteria and the questionnaires provided to the participants have been previously discussed in detail^(16,17)^.

Out of the initial 4011 HYDRIA participants, SFL measurement was restricted to participants above the age of 55 years due to financial constraints (*n* 748). Participants who reported use of iron-containing medication, defined as iron preparations (B03A) within the Anatomical Therapeutic Chemical classification system^(18)^ and dietary supplements of any kind, were excluded from the analysis, since consumption of such preparations could suggest a relevant health condition that would introduce bias to the analysis. Thus, the remaining sample size was 517 participants. The HYDRIA survey was approved by the Hellenic Health Foundation’s Bioethics Committee in accordance with the Declaration of Helsinki. All participants provided their informed written consent prior to their inclusion in the study.

### Biochemical analysis

The blood samples were analysed by ‘Bioiatriki’ Healthcare Group. The electrochemiluminescence immunoassay, ECLIA, was used to quantify SFL. The method used for CRP was nephelometry, and high-sensitivity CRP was measured.

### Dietary assessment

Dietary intake was assessed using two non-consecutive 24-h dietary recalls (24-HDR) per participant, with a time interval of approximately 15–30 d between them. During the 24-HDR, participants recalled their food and beverage intake and answered questions, among others, about the food preparation and the amounts consumed, using the Hellenic Health Foundation Nutrition Tool.

The adherence to the MD pattern was assessed using a 9-point Mediterranean diet score (MDS). For the estimation of MDS, each of the nine dietary components was scored 0 or 1 based on sex-specific median intakes. Vegetables, legumes, fruits and nuts, cereals and fish scored 1 for intakes at or above the median and 0 otherwise; meat and dairy products scored 1 for intakes below the median and 0 otherwise. Alcohol scored 1 for moderate consumption (10–50 g/d for men, 5–25 g/d for women). The monounsaturated:saturated fat ratio was used to assess fat intake (lipids). The total MDS ranged from 0 (minimal adherence) to 9 (maximal adherence)^(19)^.

### Statistical analysis

The association between SFL (ng/ml) with adherence to MD, dietary iron intake and other potential predictors was examined using linear regression models. In these models, SFL was log-transformed to better approach normality. The prevalence of ID, defined as SFL lower than 15 ng/ml and 30 ng/ml, was estimated overall, as well as by age and sex. SFL were adjusted for inflammation according to the BRINDA (Biomarkers Reflecting Inflammation and Nutritional Determinants of Anaemia) method, using only CRP, since alpha-1-acid glycoprotein was not measured in the study^(20)^. Potential predictors of ID were also examined, in this case by logistic regression models for both thresholds.

MDS was categorised as low (0–3), intermediate (4–6) and high (7–9). Total dietary iron intake (mg/d) and dietary iron intake from haem iron sources, that is, red meat, fish and poultry (haem iron, mg/d), were also examined as predictors of SFL and ID. The dietary iron intake for each participant was estimated with the use of the mean of their two 24-HDR. Haem iron intake was estimated by considering 40 % of the total iron in meat, poultry and fish as haem iron, based on previously described methodology.^(21)^ To remove confounding by total energy intake, account for inter-individual differences in food consumption and mitigate the impact of dietary misreporting associated with 24-HDR, iron intake was energy-adjusted to 2000 kcal/d, the standard reference for adults, using the nutrient-density method (absolute nutrient intake divided by total energy)^(22,23)^.

For the evaluation of the daily iron requirement, the EFSA population reference intake was used, that is, the intake level that meets the needs of 97·5 % of the individuals in the population, which is 11 mg/dl for males and postmenopausal females^(24)^.

Further potential predictors for both linear and logistic regression models included sex; age grouped by 10 years (55–64, 65–74, 75+); BMI (< 25, 25 to < 30, equal or > 30); level of education: low (≤ 9 years of formal education), intermediate (> 9 to ≤ 12 years) and high (> 12 years, including those with a bachelor’s degree or higher)^(25)^; cigarette smoking (current smoker, former smoker, non-smoker); and high-sensitivity CRP. To account for skewness, high-sensitivity CRP was log-transformed when entered in regression models. The categories of ‘low’ and ‘within normal limits’ BMI (BMI < 25) comprised one larger category due to the very low number of participants with a BMI below the normal level (BMI < 18·5). Other than that, the cutoff values to determine the BMI categories followed the respective WHO recommendations^(26)^. Physical activity was calculated in metabolic equivalents of task (MET), representing the ratio of energy expended in an activity to that when resting. Daily physical activity was calculated by multiplying the duration (h/d) of each reported activity by its corresponding MET value and adding the cumulative MET-hours across all activities^(27)^.

All analysis has been performed considering HYDRIA’s two-stage, stratified random sampling design. The crude associations between SFL and ID and each predictor were examined by weighted univariable linear and logistic regression models. Multivariable analyses were also considered.

Level of significance was set at *α* equal to 0·05. The statistical analysis was performed with STATA/se 11·0 (StataCorp, College Station).

## Results

The sample includes 517 individuals, of whom 54·1 % men. The majority of the participants were overweight (37·1 %) or obese (44·9 %), and 73 % of the participants had a low educational level, while 46·6 % of them were non-smokers. The adherence to the MD paradigm was mostly intermediate (43 %). Mean CRP levels were 3·7 mg/l, and 84·8 % of the participants had normal CRP levels (i.e. < 5 mg/l). The mean dietary iron intake in mg/d was 10·7 and 1·6 for total iron and haem iron, respectively (Table 1).

**Table 1.** Counts (*n*) and percentages (%) by categorical variables’ levels and mean values and sd for the continuous variables of the 517 study participants Table 1 long description.

	Total (*n* 517)		Women (*n* 228)		Men (*n* 289)		*P*
	*n*	%	*n*	%	*n*	%	
Age groups							0·343
55–64 years	241	39	104	35·8	137	41·6	
65–74 years	167	29·1	76	27·3	91	30·6	
> 74 years	109	31·9	48	36·8	61	27·8	
BMI							0·287
Underweight/normal	59	11·3	23	9·6	36	12·8	
Overweight	203	37·1	79	33·1	124	40·6	
Obese	221	44·9	117	52·6	104	38·4	
Unknown	34	6·6	9	4·7	25	8	
MDS							0·893
Low (0–3)	175	34·5	84	36·5	91	32·8	
Intermediate (4–5)	229	43	100	43·1	129	42·9	
High (6–9)	98	20·6	41	19·6	57	21·5	
Unknown	15	1·9	3	0·8	12	2·9	
Smoking							< 0·001
Daily smoker	120	21·3	45	16·3	75	25·5	
Former smoker	166	30·4	36	12·6	130	45·5	
Non-smoker	217	46·6	144	70·4	73	26·4	
Unknown	14	1·8	3	0·8	11	2·6	
Educational level							0·012
Low	274	73	137	79·5	137	67·5	
Intermediate	130	16	53	13·7	77	18	
High	113	11	38	6·9	75	14·5	
Iron intake, total (mg/d)							< 0·001
< 11	274	57·2	161	77·4	113	40	
>= 11	228	40·9	64	21·8	164	57	
Unknown	15	1·9	3	0·8	12	2·9	
	Mean	sd	Mean	sd	Mean	sd	
Physical activity (MET-h/d), *n* 503	42·1	8·5	43·1	7·6	41·2	9·1	0·121
CRP (mg/l), *n* 506	3·7	7·5	3·4	4·3	4	9·5	0·521
Dietary iron intake (total, non-energy adjusted) (mg/d), *n* 502	10·7	5·5	8·6	4·2	12·4	5·9	< 0·001
Dietary haem iron (non-energy adjusted) (mg/d), *n* 502	1·6	2·3	1·5	2·5	1·7	2·2	0·524
Dietary iron intake (total, energy-adjusted) (mg/d), *n* 502	13·3	4·5	13·3	4·6	13·3	13·3	0·945
Dietary haem iron (energy-adjusted) (mg/d), *n* 502	1·9	2·6	2	3·1	1·8	1·8	0·472

MDS, Mediterranean diet score; MET, metabolic equivalents of task; CRP, C-reactive protein.

After BRINDA adjustment, mean SFL was 117 ng/ml overall (men: 142 ng/ml, women: 88 ng/ml), while the effect of age was different in men and women (age and sex interaction *P*-value < 0·001). Men demonstrated, on average, a more prominent and consistent decrease of SFL with increased age, with a drop of 38·4 % in the age group of 75+ (*P* = 0·008) compared with the 55–64 age group. Women had, on average, the highest SFL in the age group of 65–74 years, although this difference was not statistically significant when compared with women aged 55–64 years (*P* = 0·881). Above the age of 74, women experienced a drop of 44·2 %, compared with women 55–64 years old (*P* = 0·025). SFL were similar across the levels of adherence to the MD (*P*-value = 0·333). No association was observed between SFL and dietary total or haem iron intake. (Table 2).

**Table 2.** Mean SFL in participants’ subgroups and mean change of SFL for one-unit increase of the continuous characteristics Table 2 long description.

	SFL (ng/ml)			
	*n*	Mean	sd	P^*^
Covariate				
Age, women^†^				0·039
55–64 years	102	94·5	67·8	
65–74 years	74	112·9	131·9	0·881
> 74 years	48	63	54·4	0·025
Age, men^†^				0·026
55–64 years	134	174·7	123·9	
65–74 years	87	129·7	84·9	0·117
> 74 years	61	107·6	103·7	0·008
MDS				0·349
Low (0–3)	168	112·1	115·7	
Intermediate (4–5)	225	121·3	96·2	0·146
High (6–9)	98	117·7	106·4	0·316
BMI				0·655
Underweight/normal	56	102·7	77·5	
Overweight	197	123·6	102·4	0·436
Obese	219	109·4	92·8	0·906
Smoking				0·156
Daily smoker	104	125·6	112·5	
Occasionally smoker	10	139·9	129·5	0·354
Former smoker	166	132·1	107·8	0·716
Non-smoker	212	103·4	98·2	0·226
Educational level				0·172
Low	269	113·3	102·9	
Intermediate	127	140·7	127·3	0·061
High	110	106·6	72·5	0·436
		Mean change of SFL	95 % CI	
Physical activity (MET-h/d)	492	0·01	−0·007, 0·028	0·249
Dietary iron intake (total, non-energy adjusted)	491	0·03	−0·02, 0·07	0·222
Dietary haem iron intake (non-energy adjusted)	491	−0·001	−0·06, 0·06	0·974
Dietary iron intake (total, energy-adjusted)	491	−0·004	−0·05, 0·04	0·843
Dietary haem iron intake (energy-adjusted)	491	−0·02	−0·07, 0·03	0·482

MDS, Mediterranean diet score; SFL, serum ferritin levels; MET, metabolic equivalents of task.

* *P*-values are estimated by weighted linear regression.

† Age by sex interaction *P*-value: < 0·001.

SFL < 30 ng/ml was observed in 12·7 % of the total population (9·2 % of men, 16·7 % of women), while SFL < 15 ng/ml was present in 6·5 % overall (4·0 % of men, 9·4 % of women). Two separate logistic regression models were fitted to identify significant predictors of ID using both thresholds (Table 3). Unlike the case of SFL, the age and sex interaction was not significant in either the logistic regression models (*P*-values: 0·272 and 0·213 for SFL < 30 ng/ml and SFL < 15 ng/ml, respectively).

**Table 3.** Results from the weighted logistic regression models for SFL < 30 ng/ml and SFL < 15 ng/ml Table 3 long description.

		SFL < 30 ng/ml			SFL < 15 ng/ml		
	*n*	OR	95 % CI	*P*	OR	95 % CI	*P*
Covariate							
Sex^*^				0·178			0·256
Women	224	1	1·00, 1·00		1	1·00, 1·00	
Men	282	0·5	0·19, 1·37	0·178	0·4	0·08, 1·96	0·256
Age groups (total)^*^				0·086			0·062
55–64 years	236	1	1·00, 1·00		1	1·00, 1·00	
65–74 years	161	1·6	0·49, 5·19	0·430	0·88	0·13, 6·07	0·894
> 74 years	109	3·87	1·14, 13·08	0·030	5·6	1·08, 29·16	0·041
MDS				0·074			0·007
Low (0–3)	168	1	1·00, 1·00		1	1·00, 1·00	
Intermediate (4–5)	225	0·24	0·07, 0·87	0·031	0·1	0·02, 0·41	0·002
High (6–9)	98	0·32	0·07, 1·55	0·157	0·4	0·05, 2·91	0·359
BMI				0·678			0·722
Underweight/normal	56	1	1·00, 1·00		1	1·00, 1·00	
Overweight	197	0·47	0·08, 2·66	0·387	1	0·04, 23·92	0·998
Obese	219	0·8	0·22, 2·87	0·725	1·99	0·19, 20·74	0·559
Smoking				0·574			0·272
Current smoker	114	1	1·00, 1·00		1	1·00, 1·00	
Former smoker	166	1·41	0·33, 5·98	0·636	0·59	0·07, 4·76	0·614
Non-smoker	212	1·98	0·52, 7·57	0·315	2·19	0·31, 15·59	0·431
Educational level				0·103			0·183
Low	269	1	1·00, 1·00		1	1·00, 1·00	
Intermediate	127	0·51	0·24, 1·06	0·071	0·21	0·04, 1·10	0·065
High	110	0·44	0·20, 0·97	0·043	0·65	0·21, 2·00	0·453
Physical activity (MET-h/d)	492	0·99	0·93, 1·05	0·739	0·96	0·87, 1·06	0·436
Dietary iron intake (total, non-energy adjusted)	491	0·97	0·79, 1·18	0·731	0·86	0·65, 1·14	0·290
Dietary haem iron intake (non-energy adjusted)	491	1·04	0·85, 1·28	0·674	0·94	0·72, 1·23	0·638
Dietary iron intake (total, energy-adjusted)	491	1	0·84, 1·19	0·999	0·87	0·61, 1·24	0·439
Dietary haem iron intake (energy-adjusted)	491	1·06	0·91, 1·22	0·469	1·01	0·83, 1·22	0·951

SFL, serum ferritin levels; MDS, Mediterranean diet score; MET, metabolic equivalents of task.

* Age by sex interaction *P*-value: 0·272 and 0·213 for SFL < 30 ng/ml and SFL < 15 ng/ml, respectively.

The overall effects of sex and age on ID were not statistically significant for either threshold (SFL < 30 or < 15 ng/ml) in the total population. Nevertheless, individuals > 74 years old had 5·6 times and 3·9 times increased odds for SFL < 15 ng/ml (*P* = 0·041) and for SFL < 30 ng/ml (*P* = 0·030, respectively, compared with 55–64 years old individuals. No significant differences were observed for the 65–74 years age group.

Overall, adherence to MD was not found to be significantly associated with SFL < 30 ng/ml (*P* = 0·074), although intermediate adherence was associated with decreased odds for SFL < 30 ng/ml (OR = 0·24; 95 % CI 0·07, 0·87; *P* = 0·031) and a similar but non-significant association was observed for those with high adherence (OR = 0·32; 95 % CI 0·07, 1·55; *P* = 0·157). When ID was defined as SFL < 15 ng/ml, MD adherence was significantly associated with lower odds of ID (*P* = 0·007), mainly due to the effect of intermediate adherence (OR = 0·10; 95 % CI 0·02, 0·41; *P* = 0·002) but not high adherence. These associations remained significant in the presence of age and sex. Dietary indicators of iron intake were not found to have a significant effect on SFL or ID (Table 3).

Dietary iron intake was significantly lower in women (men: 12·4 mg/d *v*. women 8·6 mg/d, *P* < 0·001) and decreased significantly with increased age (*P* = 0·013 for 65–74 and *P* < 0·001 for > 75, both *v*. 55–64), especially in men. Thus, a significant sex by age interaction was observed. Specifically, the difference between men and women was greater in the 55–64 age group and was attenuated with advancing age (*P* < 0·001) (online Supplementary Table S1). Energy-adjusted (2000 kcal) total iron intake was significantly higher in the highest adherence category. In contrast, non-adjusted haem iron intake was lower in the highest MD adherence group, a trend that persisted in the energy-adjusted values, although the latter was not statistically significant (*P* = 0·057) (online Supplementary Table S2).

## Discussion

To our knowledge, this is the first study in Greece that evaluates iron status and influencing factors in a large sample of participants of a nationally representative sample. Among the population, 6·5 % had ID, with a mean SFL of 117 ng/ml. SFL were significantly associated with sex and age, presenting lower levels in women, with individuals > 74 years old showing higher odds of ID at both thresholds, compared with those aged 55–64 years. 57·2 % of the population had dietary iron intake below the EFSA population reference intake of 11 mg/d. Dietary iron intake declined considerably with increased age and was significantly lower in women. Intermediate MD adherence was significantly associated with a reduced risk of ID at both thresholds.

Few studies have explored iron status and adequacy in specific subpopulations residing in Greece, mainly children^(28,29)^ or army recruits^(30)^ or other subpopulations where iron metabolism was estimated^(31)^.

Overall, the mean SFL (117 ng/ml) is within normal levels for both sexes. The observed prevalence of ID indicates that ID is not extensive in the older Greek population. It is difficult to compare iron status among different countries due to differences in methodology, cutoff values, biomarkers used (i.e. SFL, serum iron levels, Hb, soluble transferrin receptor and total body iron) and age groups. Indicatively, in Slovenia, only 0·6 %^(32)^ of the older population had SFL < 15 μg/l, and in Portugal, 17 %^(33)^ of the older population had SFL < 15 μg/l. In the older population of the USA, the prevalence of ID was 10 %^(34)^, while in a study that included community-dwelling older adults from five European countries, it was 4·2 % based on SFL < 30 μg/l^(5)^. The mean value of SFL is in accordance with studies of similar age groups in various countries^(32,35,36)^.

SFL were significantly lower in women and declined with increasing age. Iron accumulation is thought to vary by age, sex and between pre- and postmenopausal women^(37)^. Studies have shown that hepcidin, a hormone that regulates iron metabolism in the body, is lower in women than in men and higher in postmenopausal women than in those of reproductive age, with the hypothesis that oestrogen levels alter hepcidin levels^(37,38)^. Lower SFL in women compared with men in our study are in accordance with other studies in the literature^(39)^. The decreasing trend of SFL with advancing age was more evident in men. While older men present a drop in SFL, older women after menopause present a rise^(37)^. Menstrual cessation results in reduced iron losses, which raises iron stores^(37)^. Both sexes present a sharper decline in SFL after the age of 74 years old. This drop may be attributed to poor iron intake or lower absorption^(40)^. For the age group > 74 years old, there were considerably increased odds for ID compared with the 55–64 age group.

In our study, we did not find a significant association of SFL with BMI. Conversely, several studies have shown a link between obesity and ID, which is prominent in all age groups^(41)^. The most probable cause is obesity-related inflammation as studies indicate no differences in dietary iron intake between the obese and non-obese individuals^(42)^, but rather regarding iron absorption. Inflammation increases hepcidin, which blocks iron release from reticuloendothelial cells and decreases intestinal iron absorption^(43)^.

Infection and inflammation have an impact on several nutritional biomarkers, including iron. Inflammation results in the allocation of iron in favour of storage sites, including iron retention by macrophages and liver, resulting in decreased serum iron and increased SFL^(44)^. WHO advises measuring another acute-phase reactive protein, such as CRP, along with SFL to examine the presence of inflammation^(2)^. Following this argument, in our analysis, SFL were adjusted for inflammation using the BRINDA method. Furthermore, since almost half of our population was obese, the obesity-related inflammation underscores the need to adjust SFL for inflammation to prevent underestimation of ID.

57·2 % of the population presented inadequate daily iron intake based on the EFSA population reference intake (11 mg/d), with a mean dietary intake of 10·7 mg/d (men 12·3 mg/d; women 8·9 mg/d). Iron intakes were lower than in the Hellenic National Nutrition and Health Survey (HNNHS), which reported median iron intakes of 15 mg/d and 11·6 mg/d for men and 13·3 mg/d and 11·5 mg/d for women in the 51–70 and > 70-year age groups, and was conducted during the same period as HYDRIA^(45)^. Dietary iron intake ranges from 10·2 to 16 mg/d across other national representative studies^(32,35,46)^. Dietary iron intake declined with increased age in both sexes, but men presented higher values. A higher proportion of men had adequate iron intake compared with women. In women, the mean dietary iron intake was below the population reference intake of 11 mg/d across all age groups, whereas only men over 74 years had intake below the reference value. According to EFSA, men in general present to some extent greater average daily intakes than women, primarily because they consume bigger amounts of food each day^(24)^.

In this survey, the intake of haem iron accounted for 15·9 % of unadjusted total dietary iron. Previous findings from HYDRIA showed that men had higher consumption of products with haem iron (meat, poultry, etc.)^(17)^. Dietary haem iron was not significantly associated with SFL. This is in contrast with other published studies where haem iron is an independent factor for iron status^(47,48)^, although there are studies that are in accordance with ours^(49,50)^.

Non-haem iron was not presented in our study as its absorption depends on individual iron stores and is strongly influenced by dietary enhancers and inhibitors, making reported intake an unreliable marker of actual absorption^(12)^. In any case, non-haem iron intake (total iron minus haem iron) had a non-significant effect on BRINDA-adjusted SFL (*β* = 3·02; 95 % CI –0·08, 6·12; *P* = 0·086) and did not affect overall results, so it was not included as a primary finding.

Additionally, the MDS was evaluated in our cohort. Total iron intake, after being adjusted to a 2000 kcal diet, was significantly higher among individuals with the highest MD adherence. The unadjusted values demonstrated a similar pattern, but the difference was not statistically significant. There are a few studies that explore the relationship between adherence to MD and iron intake. Our results confirm previous studies that explored the same issue, namely, that there is a positive association regarding adherence to MD and total iron intake in the older population^(51–53)^. Higher adherence to the MD has also been associated with lower iron inadequacy in children and adolescents^(11)^. Adherence to MD did not significantly affect mean SFL. Although the intake of highly bioavailable haem iron decreased with increasing adherence to the MD, iron status did not differ significantly. This finding implies that the MD offers enough bioavailable iron despite lower haem iron. Its combination of plant-based iron sources and dietary enhancers (such as vitamin C) possibly improves non-haem iron absorption^(51,54)^. In a study with male adolescents, iron absorption was enhanced when a Mediterranean-based diet was consumed^(54)^. Conversely, a study that included older men from Crete, where MD used to prevail, has demonstrated lower SFL compared with men from Zutphen in the Netherlands^(55)^.

It is noteworthy that in our study, adherence to MD was associated with decreased odds for ID, considering SFL < 15 ng/ml. The intermediate category of MD adherence had a statistically significant protective impact against low SFL with both cutoffs. Since the effect of iron intake was not significant for ID, further studies are needed to explore this tendency. The associations observed may partly reflect heterogeneity in the dietary compounds contributing to the MDS. Individuals with similar MDS values may achieve comparable scores through different combinations of foods. For instance, some may consume higher amounts of fish and fruits, while others may emphasise cereals and legumes. Such variability may differentially affect iron intake and bioavailability, given the varying contributions of non-haem iron sources and dietary enhancers or inhibitors of iron absorption^(12)^. Additionally, in the HYDRIA study, higher MD adherence was more prevalent among older participants^(17)^, who may experience reduced intestinal iron absorption, potentially attenuating the expected benefits of higher adherence levels.

This study presents several strengths and limitations. Its major strength is that the sample is drawn from a national representative study, allowing the findings to be generalised to the population of older persons who do not use iron medication or supplements. There is an abundance of carefully selected and recorded characteristics, such as sociodemographic and lifestyle factors, and blood samples were collected from the entire population > 55 years old in our study. Moreover, dietary intake was based on 2 × 24-HDR, which is considered the appropriate and more accurate approach according to the recommendations of the European Health Examination Survey and EFSA. Additionally, to account for the potential impact of chronic low-grade inflammation on SFL and correctly estimate its levels, particularly in older adults and the obese population, the BRINDA method was applied. Nevertheless, limitations should be noted. Although SFL, according to WHO, is a reliable indicator of iron stores and can be utilised to detect ID, additional indicators of iron status, such as soluble transferrin receptor and transferrin saturation, were not available. Furthermore, the contribution of individual food groups to total iron intake was not assessed, nor were dietary factors that could affect iron absorption, including potential enhancers (i.e. vitamin C) and inhibitors (i.e. phytates and polyphenols). Also, since it is a cross-sectional study, causal associations cannot be established.

### Conclusion

In conclusion, the number of older adults who are iron deficient in a large Greek sample is low. There was a clear trend for increased risk of ID with age. There was no association between iron status and dietary iron intake, nor intake of haem iron. Adherence to the MD was associated with lower odds for ID but had no effect on SFL. Given that ID is a public health concern, further research is needed to determine the components of dietary iron consumption and its relation to iron biomarkers to quantify both the enhancers and inhibitors of iron absorption and to identify the most suitable biomarker to reflect the iron status of specific demographic groups, such as the older population.

## Supporting information

Papatesta et al. supplementary material Papatesta et al. supplementary material

## Supplementary material

For supplementary material/s referred to in this article, please visit https://doi.org/10.1017/S0007114526106540.

## Table 1 Long description

The table presents counts and percentages by categorical variables levels and mean values and standard deviations for continuous variables of 517 study participants. It includes data on age groups, BMI, Mediterranean Diet Score (MDS), smoking status, educational level, and iron intake. The table is divided into columns for total participants, women, and men, with a P-value indicating statistical significance. Row 1: Age groups 55-64 years, 167 participants, 29.1 percent, 76 women, 27.3 percent, 91 men, 30.6 percent. Row 2: Age groups 65-74 years, 241 participants, 39 percent, 104 women, 35.8 percent, 137 men, 41.6 percent. Row 3: Age groups over 74 years, 109 participants, 31.9 percent, 48 women, 36.8 percent, 61 men, 27.8 percent. Row 4: BMI Underweight/normal, 59 participants, 11.3 percent, 23 women, 9.6 percent, 36 men, 12.8 percent. Row 5: BMI Overweight, 203 participants, 37.1 percent, 79 women, 33.1 percent, 124 men, 40.6 percent. Row 6: BMI Obese, 221 participants, 44.9 percent, 117 women, 52.6 percent, 104 men, 38.4 percent. Row 7: BMI Unknown, 34 participants, 6.6 percent, 9 women, 4.7 percent, 25 men, 8 percent. Row 8: MDS Low (0-3), 175 participants, 34.5 percent, 84 women, 36.5 percent, 91 men, 32.8 percent. Row 9: MDS Intermediate (4-5), 229 participants, 43 percent, 100 women, 43.1 percent, 129 men, 42.9 percent. Row 10: MDS High (6-9), 98 participants, 20.6 percent, 41 women, 19.6 percent, 57 men, 21.5 percent. Row 11: MDS Unknown, 15 participants, 1.9 percent, 3 women, 0.8 percent, 12 men, 2.9 percent. Row 12: Smoking Daily smoker, 120 participants, 21.3 percent, 45 women, 16.3 percent, 75 men, 25.5 percent. Row 13: Smoking Former smoker, 166 participants, 30.4 percent, 36 women, 12.6 percent, 130 men, 45.5 percent. Row 14: Smoking Non-smoker, 217 participants, 46.6 percent, 144 women, 70.4 percent, 73 men, 26.4 percent. Row 15: Smoking Unknown, 14 participants, 1.8 percent, 3 women, 0.8 percent, 11 men, 2.6 percent. Row 16: Educational level Low, 274 participants, 73 percent, 137 women, 79.5 percent, 137 men, 67.5 percent. Row 17: Educational level Intermediate, 130 participants, 16 percent, 53 women, 13.7 percent, 77 men, 18 percent. Row 18: Educational level High, 113 participants, 11 percent, 38 women, 6.9 percent, 75 men, 14.5 percent. Row 19: Iron intake, total (mg/d) < 11, 274 participants, 57.2 percent, 161 women, 77.4 percent, 113 men, 40 percent. Row 20: Iron intake, total (mg/d) >= 11, 228 participants, 40.9 percent, 64 women, 21.8 percent, 164 men, 57 percent. Row 21: Iron intake Unknown, 15 participants, 1.9 percent, 3 women, 0.8 percent, 12 men, 2.9 percent. Row 22: Physical activity (MET-h/d), Mean 42.1, SD 8.5, Mean 43.1, SD 7.6, Mean 41.2, SD 9.1. Row 23: CRP (mg/l), Mean 3.7, SD 7.5, Mean 3.4, SD 4.3, Mean 4, SD 9.5. Row 24: Dietary iron intake (total, non-energy adjusted) (mg/d), Mean 10.7, SD 5.5, Mean 8.6, SD 4.2, Mean 12.4, SD 5.9. Row 25: Dietary haem iron (non-energy adjusted) (mg/d), Mean 1.6, SD 2.3, Mean 1.5, SD 2.5, Mean 1.7, SD 2.2. Row 26: Dietary iron intake (total, energy-adjusted) (mg/d), Mean 13.3, SD 4.5, Mean 13.3, SD 4.6, Mean 13.3, SD 4.5. Row 27: Dietary haem iron (energy-adjusted) (mg/d), Mean 1.9, SD 2.6, Mean 2, SD 3.1, Mean 1.8, SD 1.8.

Navigate back to Table 1.

## Table 2 Long description

A table with 12 rows and 5 columns comparing mean serum ferritin levels (SFL) in participants subgroups and mean change of SFL for one-unit increase of the continuous characteristics. The columns are labeled Covariate, n, Mean, SD, and P*. The table includes data for age groups for women and men, Mediterranean Diet Score (MDS), Body Mass Index (BMI), smoking status, educational level, physical activity, and dietary iron intake. Each row provides the number of participants (n), mean SFL levels in ng/ml, standard deviation (SD), and P-values for different subgroups. The table shows variations in SFL levels across different age groups, dietary scores, BMI categories, smoking habits, educational levels, physical activity, and dietary iron intake.

Navigate back to Table 2.

## Table 3 Long description

A table comparing logistic regression models for SFL < 30 ng/ml and SFL < 15 ng/ml. The table has 12 rows and 10 columns. Column headers are Covariate, n, OR, 95% CI, P for SFL < 30 ng/ml, OR, 95% CI, P for SFL < 15 ng/ml. Row labels include Sex, Age groups (total), MDS, BMI, Smoking, Educational level, Physical activity (MET-h/d), Dietary iron intake (total, non-energy adjusted), Dietary haem iron intake (non-energy adjusted), Dietary iron intake (total, energy-adjusted), Dietary haem iron intake (energy-adjusted). Row 1: Sex, Women, 224, 1, 1.00, 1.00, 0.178, 1, 1.00, 1.00, 0.256. Row 2: Sex, Men, 282, 0.5, 0.19, 1.37, 0.178, 0.4, 0.08, 1.96, 0.256. Row 3: Age groups (total), 55-64 years, 236, 1, 1.00, 1.00, 0.086, 1, 1.00, 1.00, 0.062. Row 4: Age groups (total), 65-74 years, 161, 1.6, 0.49, 5.19, 0.430, 0.88, 0.13, 6.07, 0.894. Row 5: Age groups (total), > 74 years, 109, 3.87, 1.14, 13.08, 0.030, 5.6, 1.08, 29.16, 0.041. Row 6: MDS, Low (0-3), 168, 1, 1.00, 1.00, 0.074, 1, 1.00, 1.00, 0.007. Row 7: MDS, Intermediate (4-5), 225, 0.24, 0.07, 0.87, 0.031, 0.1, 0.02, 0.41, 0.002. Row 8: MDS, High (6-9), 98, 0.32, 0.07, 1.55, 0.157, 0.4, 0.05, 2.91, 0.359. Row 9: BMI, Underweight/normal, 56, 1, 1.00, 1.00, 0.678, 1, 1.00, 1.00, 0.722. Row 10: BMI, Overweight, 197, 0.47, 0.08, 2.66, 0.387, 1, 0.04, 23.92, 0.998. Row 11: BMI, Obese, 219, 0.8, 0.22, 2.87, 0.725, 1.99, 0.19, 20.74, 0.559. Row 12: Smoking, Current smoker, 114, 1, 1.00, 1.00, 0.574, 1, 1.00, 1.00, 0.272. Row 13: Smoking, Former smoker, 166, 1.41, 0.33, 5.98, 0.636, 0.59, 0.07, 4.76, 0.614. Row 14: Smoking, Non-smoker, 212, 1.98, 0.52, 7.57, 0.315, 2.19, 0.31, 15.59, 0.431. Row 15: Educational level, Low, 269, 1, 1.00, 1.00, 0.103, 1, 1.00, 1.00, 0.183. Row 16: Educational level, Intermediate, 127, 0.51, 0.24, 1.06, 0.071, 0.21, 0.04, 1.10, 0.065. Row 17: Educational level, High, 110, 0.44, 0.20, 0.97, 0.043, 0.65, 0.21, 2.00, 0.453. Row 18: Physical activity (MET-h/d), 492, 0.99, 0.93, 1.05, 0.739, 0.96, 0.87, 1.06, 0.436. Row 19: Dietary iron intake (total, non-energy adjusted), 491, 0.97, 0.79, 1.18, 0.731, 0.86, 0.65, 1.14, 0.290. Row 20: Dietary haem iron intake (non-energy adjusted), 491, 1.04, 0.85, 1.28, 0.674, 0.94, 0.72, 1.23, 0.638. Row 21: Dietary iron intake (total, energy-adjusted), 491, 1, 0.84, 1.19, 0.999, 0.87, 0.61, 1.24, 0.439. Row 22: Dietary haem iron intake (energy-adjusted), 491, 1.06, 0.91, 1.22, 0.469, 1.01, 0.83, 1.22, 0.951.

Navigate back to Table 3.
