# Supplementary material for: Serum ferritin levels in a sample of older participants in the Greek HYDRIA survey: associations with sociodemographic and lifestyle parameters including dietary iron intake and Mediterranean diet score
Source: Br J Nutr. 2026 Feb 10;135(11):1185–92. doi: 10.1017/S0007114526106540 (PMC13423517; doi:10.1017/S0007114526106540)
Supplement: Papatesta et al. supplementary material [file S0007114526106540sup001.docx]

**Table S1**. Dietary Iron Intake by sex and age group summarized as mean and standard deviation (A) and count and percentage of individuals with adequate iron intake (>11mg/day) (B) ^†^

|  | Total | | Women | | Men | | p-value* |
| --- | --- | --- | --- | --- | --- | --- | --- |
| A.(mg/day) | Mean | SD | Mean | SD | Mean | SD |  |
| 55-64 years | 12.6 | 6.6 | 9.7 | 4.8 | 14.8 | 7.0 | 0.004 |
| 65-74 years | 10.3 | 4.6 | 8.6 | 4.4 | 11.6 | 4.3 | 0.005 |
| >74 years | 8.6 | 3.6 | 7.6 | 3.0 | 9.8 | 3.9 | 0.066 |
| B. (N %) | N | % | N | % | N | % | p-value* |
| 55-64 years | 124 | 51.7 | 34 | 28.0 | 90 | 69.0 | 0.001 |
| 65-74 years | 74 | 42.2 | 21 | 19.4 | 53 | 59.5 | 0.005 |
| >74 years | 30 | 26.5 | 9 | 17.6 | 21 | 36.5 | 0.199 |

^†^Population Reference Intake (PRI) values (11 mg/day for males and postmenopausal women) according to EFSA

*****P-values are estimated by weighted linear regression (A) and weighted chi-square test (B)

**Table S2**. Total and Heme Iron Intake (Unadjusted and Energy-Adjusted) by Mediterranean Diet Score (N=502).

|  | Low (0-3) MDS | | Intermediate (4-6) MDS | | High (7-9) MDS | | p-value* |
| --- | --- | --- | --- | --- | --- | --- | --- |
|  | n=175 | | n=229 | | n=98 | |  |
| Iron intake (mg/day) | Mean | SD | Mean | SD | Mean | SD |  |
| Total iron intake, non-energy adjusted | 8.7 | 4.0 | 11.4 | 6.0 | 12.4 | 5.5 | 0.381 |
| Heme iron intake, non-energy adjusted | 1.6 | 1.7 | 1.7 | 2.9 | 1.3 | 1.8 | 0.003 |
| Total iron intake, 2000 kcal adjusted | 11.9 | 3.9 | 14.0 | 5.2 | 14.2 | 3.3 | 0.002 |
| Heme iron intake, 2000 kcal adjusted | 2.1 | 2.0 | 1.9 | 3.2 | 1.4 | 1.9 | 0.057 |

MDS, Mediterranean Diet Score

*P-values are estimated by weighted linear regression
